# Development and Validation of the Japanese Version of the Bergen Social Media Addiction Scale (BSMAS-J)

**DOI:** 10.3390/ejihpe16100148

**Published:** 2026-09-24

**Authors:** Zhixian Lian, Mengting Zhang, Zeyu Liu, Xue Dou

**Affiliations:** 1 School of Culture and Communication, Guangdong University of Business and Technology, Zhaoqing 526020, China; 2 Department of Hygiene and Preventive Medicine, Fukushima Medical University, Hikarigaoka, Fukushima 960-1295, Japan; m-zhang@fmu.ac.jp; 3 Open Innovation & Collaboration Research Organization, Ritsumeikan University, Ibaraki 567-8570, Osaka, Japan; 26v04361@gst.ritsumei.ac.jp; 4 College of Comprehensive Psychology, Ritsumeikan University, Ibaraki 567-8570, Osaka, Japan

**Keywords:** Bergen Social Media Addiction Scale (BSMAS), social media addiction, psychometric properties, reliability and validity, Japan

## Abstract

Problematic social media use (PSMU) has become a growing concern worldwide, highlighting the need for reliable and culturally valid assessment tools to examine its psychological correlates. However, comprehensive psychometric evaluations of PSMU measures in the Japanese context remain limited. The present study aimed to develop and validate the Japanese version of the Bergen Social Media Addiction Scale (BSMAS-J) among Japanese college students. A total of 314 undergraduate students completed the BSMAS-J, along with the Internet Addiction Test (IAT), the Behavioral Inhibition and Behavioral Activation Scales (BIS/BAS), and the Ten-Item Personality Inventory (TIPI-J). Of these, 257 participants completed a follow-up assessment 4 weeks later. To rigorously evaluate the factor structure, an independent split-sample cross-validation approach was employed, with exploratory factor analysis conducted on one subsample (*n* = 100) and confirmatory factor analysis on the other (*n* = 214). Results confirmed a robust unidimensional structure with good model fit, high internal consistency across waves (Cronbach’s *α* 0.89, McDonald’s *ω* 0.91), and moderate 4-week test–retest reliability (ICC = 0.65). Furthermore, evidence supported both longitudinal and multigroup sex measurement invariance. Convergent validity was established through strong positive associations with generalized internet addiction. Multiple regression analysis indicated that female sex, higher neuroticism, and elevated behavioral inhibition predicted BSMAS-J scores, highlighting potential avoidance-oriented coping mechanisms. Overall, the BSMAS-J demonstrates sound psychometric properties as a brief and reliable assessment tool, establishing a solid empirical foundation for future cross-cultural and longitudinal investigations in Japan.

## 1. Introduction

In recent years, social media platforms (e.g., X, Instagram) have expanded exponentially worldwide, becoming a dominant medium for daily interpersonal communication (Pellegrino et al., 2022; Rouleau et al., 2023). However, parallel to this rapid digital integration, uncontrolled or excessive engagement—commonly conceptualized as problematic social media use (PSMU) or social media addiction (SMA)—has emerged as a major global public health concern (Bottaro et al., 2025; Rouleau et al., 2023; Vossen et al., 2024). A substantial body of empirical literature demonstrates that PSMU is significantly associated with a wide spectrum of adverse psychological outcomes, including heightened psychological stress, depressive symptoms, anxiety, emotional agitation, reduced subjective well-being, and diminished life satisfaction (Abiddine et al., 2024; Bottaro et al., 2025; Brailovskaia & Margraf, 2024; Boniel-Nissim et al., 2022; Chang et al., 2022; Dadiotis et al., 2021; Stănculescu, 2023). Moreover, PSMU has been strongly linked to substance use, including smoking, drunkenness, and cannabis use (Bozhar et al., 2025; Zhong & Tian, 2026). Consequently, accurately assessing and screening for PSMU is imperative for better understanding its etiology and mitigating its negative psychosocial impacts.

To evaluate PSMU effectively, the availability of standardized and psychometrically robust assessment instruments is essential. Among various available scales, the Bergen Social Media Addiction Scale (BSMAS; Andreassen et al., 2016) has become one of the most widely recognized standard tools internationally (Bottaro et al., 2025; Pellegrino et al., 2022). Developed from the platform-specific Bergen Facebook Addiction Scale (BFAS; Andreassen et al., 2012), the BSMAS consists of six items designed to assess the risk of problematic use across social media platforms in general (Andreassen et al., 2016; Monacis et al., 2017). Due to its brevity, sound theoretical foundation, and robust psychometric properties, the BSMAS has been translated and validated across numerous languages and cultural contexts, including Arabic (Abiddine et al., 2024), Chinese (Leung et al., 2020), Greek (Dadiotis et al., 2021), Hungarian (Bányai et al., 2017), Italian (Monacis et al., 2017), Korean (Shin, 2022), Persian (Lin et al., 2017), Romanian (Stănculescu, 2023), and Thai (Ruckwongpatr et al., 2024). However, despite its global ubiquity, a psychometrically validated Japanese version of the BSMAS remains currently unavailable.

The validation of a Japanese version of the BSMAS (BSMAS-J) is vital not only for providing a standardized screening tool within Japan, but also for facilitating meaningful cross-cultural research. A key strength of the BSMAS lies in its empirical support for measurement invariance—a statistical prerequisite ensuring that an instrument measures the same underlying construct equivalently across diverse demographic and cultural groups (Chen, 2007; Vandenberg & Lance, 2000). Prior studies have established the measurement invariance of the BSMAS across sex (Abiddine et al., 2024; Monacis et al., 2017; Yue et al., 2022), cultures (Leung et al., 2020; Nurmala et al., 2026; Rouleau et al., 2023), and time (Gomez et al., 2024). Given Japan’s distinct socio-cultural context and patterns of digital communication, evaluating whether the BSMAS-J demonstrates measurement invariance across sex and over time could provide an important empirical foundation for subsequent cross-cultural comparisons, thereby allowing researchers to explore how socio-cultural factors influence PSMU.

To develop and validate the Japanese version of the Bergen Social Media Addiction Scale (BSMAS-J), the selection of validation measures in the present study was guided by the foundational studies of the original scales (Andreassen et al., 2012, 2016). Specifically, the Internet Addiction Test (IAT) was included as a widely adopted measure of generalized internet addiction to represent broader online behaviors (e.g., gaming, video streaming). In addition, the Behavioral Inhibition and Activation Systems (BIS/BAS) and the Japanese version of the Ten-Item Personality Inventory (TIPI-J; Oshio et al., 2012) were incorporated to examine personality and neurobehavioral traits, directly following the validation approach of the original scales while minimizing participant burden in an online survey.

Accordingly, the current study hypothesized that (1) the BSMAS-J would demonstrate a unidimensional structure, as supported by independent cross-validation; (2) the BSMAS-J would show acceptable internal consistency and adequate test–retest reliability over a 4-week interval, as well as measurement invariance across time and sex; and (3) consistent with the original foundational studies (Andreassen et al., 2012, 2016), the BSMAS-J would display similar patterns of associations, including significant correlations with the IAT, BIS/BAS, and TIPI-J, thereby supporting its convergent and criterion-related validity.

## 2. Materials and Methods

### 2.1. Participants

A total of 363 young adults were initially recruited through an online research company. Following the sampling methodology of the original scale development (Andreassen et al., 2012), the target population was restricted to college students; thus, 49 respondents who were not currently enrolled in higher education were excluded, yielding a baseline sample of 314 college students. The current study did not mechanically exclude any participants based on score cutoffs (e.g., ±3 *SD*), thereby preserving genuine variations in severity across the full distribution. Consequently, all 314 eligible college students were retained for the baseline analytic sample (*M_age_* = 20.44, *SD* = 1.49; 161 females [51.3%], 153 males [48.7%]). To evaluate test–retest reliability and longitudinal stability, a follow-up survey was administered 4 weeks later, completed by 257 participants (retention rate: 81.8%; attrition rate: 18.2%). Attrition analyses using Welch’s independent-samples t-tests and a chi-square test indicated no statistically significant differences between completers (*n* = 257) and non-completers (*n* = 57) regarding age (*t*(87.58) = −1.05, *p* = 0.295), baseline BSMAS-J scores (*t*(77.94) = 1.27, *p* = 0.207), or sex (*χ*^2^(1, *N* = 314) = 0.05, *p* = 0.832).

### 2.2. Measurements

#### 2.2.1. The Bergen Social Media Addiction Scale (BSMAS)

The Japanese version of the Bergen Social Media Addiction Scale (BSMAS-J) (Supplementary Materials) was adapted from the original scale developed by Andreassen et al. (2016). The BSMAS comprises six items based on the six core addiction components (salience, mood modification, tolerance, withdrawal, conflict, and relapse) proposed by Griffiths (2005) to assess the risk of social media addiction over the past year. Items are rated on a 5-point Likert scale ranging from 1 (very rarely) to 5 (very often), with higher total scores reflecting a greater severity of social media addiction.

The translation and cross-cultural adaptation process followed standard guidelines for scale translation (Inada, 2015). Permission was first obtained from the original author. The scale was translated from English into Japanese by the authors. A research team, comprising one faculty member in psychology and three graduate students, reviewed and refined the translated items to ensure clarity and consistency. The Japanese version was then back-translated into English by a bilingual psychology faculty member specializing in media psychology, who compared the back-translated version with the original English scale to establish semantic and conceptual equivalence of the items. Finally, cognitive debriefing was conducted with five Japanese college students to confirm that the items were clear, comprehensible, and conceptually appropriate.

#### 2.2.2. Internet Addiction Test (IAT)

The Internet Addiction Test (IAT; Young, 1998) was used to measure internet addiction. The scale consists of 20 items rated on a 5-point Likert scale ranging from 1 (never/does not apply) to 5 (always), with higher total scores indicating greater severity of internet addiction. The Japanese version of the IAT was obtained from the 2014 White Paper on Information and Communications published by the Ministry of Internal Affairs and Communications in Japan https://www.soumu.go.jp/johotsusintokei/whitepaper/ja/h26/html/nd2b0000.html (accessed on 1 October 2022). In the present study, the Cronbach’s *α* coefficient for the scale was 0.95.

#### 2.2.3. The BIS/BAS Scales

The Japanese version of the Behavioral Inhibition and Behavioral Activation Scales (BIS/BAS; Kamide & Daibo, 2005) was used to measure behavioral inhibition and activation systems. The instrument comprises 20 items divided into four subscales: Behavioral Inhibition System (BIS; 7 items) and three subscales of the Behavioral Activation System, consisting of Reward Responsiveness (BAS-RR; 7 items), Drive (BAS-D; 3 items), and Fun Seeking (BAS-FS; 3 items). All items are rated on a 4-point Likert scale ranging from 1 (strongly disagree) to 4 (strongly agree). In the present study, the Cronbach’s *α* coefficients for the subscales were 0.77 for BIS, 0.86 for BAS-RR, 0.77 for BAS-D, and 0.76 for BAS-FS.

#### 2.2.4. The Ten-Item Personality Inventory (TIPI-J)

To assess Big Five personality traits while reducing participant burden and enhancing data quality in an online survey format, the Japanese version of the Ten-Item Personality Inventory (TIPI-J; Oshio et al., 2012) was used rather than longer instruments. The TIPI-J comprises 10 items, with two items assessing each of the five personality dimensions: Extraversion, Agreeableness, Conscientiousness, Neuroticism, and Openness. Items are rated on a 7-point Likert scale ranging from 1 (strongly disagree) to 7 (strongly agree). Because each dimension consists of only two items, reliability was evaluated using inter-item correlations and the Spearman–Brown prophecy formula: Extraversion (*r* = 0.452, *ρ* = 0.623), Agreeableness (*r* = 0.196, *ρ* = 0.328), Conscientiousness (*r* = 0.368, *ρ* = 0.538), Neuroticism (*r* = 0.336, *ρ* = 0.503), and Openness (*r* = 0.348, *ρ* = 0.516), consistent with the psychometric properties reported by Oshio et al. (2012).

### 2.3. Procedure

Participants were recruited from an online research company in Japan (Freeasy Inc., Osaka, Japan) under the survey title “Survey on Social Media Use.” To screen for active social media users, usage frequency across major platforms (X, Facebook, Instagram, YouTube, TikTok, and online bulletin boards) was evaluated, excluding those who used social media less than once a week or not at all. Eligible participants provided electronic informed consent via the survey website and completed the first survey, in which questionnaires were presented in the following order: BSMAS-J, IAT, BIS/BAS, and TIPI-J. Demographic information, including age and sex, was also collected. Participants received compensation upon completion.

Four weeks after the first survey, a follow-up invitation was extended to a subset of participants drawn specifically from those who had completed the first survey. Those who agreed accessed the web link, completed the BSMAS-J a second time for test–retest analysis, and received additional compensation upon completion.

### 2.4. Data Analysis

Statistical analyses were conducted using R version 4.5.0, utilizing the packages psych, irr, lavaan (Rosseel, 2012), sandwich, and apaTables (Stanley, 2021). Descriptive statistics and participant attrition were first evaluated. To prevent sample-overlap bias and provide rigorous cross-validation, the baseline sample (*N* = 314) was split into two independent subsamples using sex-stratified random sampling: an EFA calibration sample (*n* = 100; 51 females, 49 males) and an independent CFA validation sample (*n* = 214; 110 females, 104 males). For the EFA (*n* = 100), factor retention was determined using parallel analysis based on polychoric correlation matrices with 5000 simulated datasets, followed by minimum residual (MinRes) factor extraction. For the independent CFA (*n* = 214), the unidimensional factor model was evaluated using robust maximum likelihood (MLR) estimation, treating the 5-point Likert items as continuous indicators. As a sensitivity analysis, the models were also examined using diagonally weighted least squares (WLSMV) with theta parameterization. Model fit was evaluated using the robust comparative fit index (CFI ≥ 0.95), robust Tucker–Lewis index (TLI ≥ 0.95), robust root mean square error of approximation (RMSEA ≤ 0.06 with 90% confidence intervals), and standardized root mean square residual (SRMR ≤ 0.08) (Hu & Bentler, 1998, 1999). Scale reliability was assessed using Cronbach’s α, McDonald’s ω, and corrected item–total correlations at both waves. Test–retest reliability across the 4-week interval (*n* = 257) was evaluated using a two-way mixed-effects, absolute-agreement, single-measurement intraclass correlation coefficient [ICC(A,1)]. Measurement invariance was evaluated sequentially across sex (*N* = 314) and across the 4-week interval (*n* = 257) using multigroup and longitudinal CFA, establishing configural, metric, and scalar invariance. In the longitudinal models, autocovariances between the same item residuals across time were specified. Invariance was supported if changes in fit indices met the recommended criteria: ∆CFI ≥ −0.010, ∆RMSEA ≤ 0.015, and ∆SRMR ≤ 0.010 (Chen, 2007). Finally, Pearson’s correlations and multiple linear regression analyses (with both ordinary least squares and HC3 robust standard errors) were performed to evaluate convergent validity and predictive relationships.

## 3. Results

### 3.1. Participant Flow and Demographic Characteristics

The participant recruitment flow and detailed demographic characteristics of the baseline sample (*N* = 314) and the 4-week follow-up sample (*n* = 257) are summarized in Table 1. Across both waves, all respondents were unmarried and without children, representing diverse residential arrangements and annual household income brackets. Demographic distributions remained highly consistent between baseline and follow-up, suggesting limited evidence of selective attrition based on these characteristics.

### 3.2. Exploratory Factor Analysis (EFA)

An exploratory factor analysis (EFA) was conducted on the randomized calibration subsample (*n* = 100). The data demonstrated high sampling adequacy, with a Kaiser–Meyer–Olkin (KMO) measure of 0.817 and a statistically significant Bartlett’s test of sphericity (*χ*^2^(15) = 508.41, *p* < 0.001). A parallel analysis based on polychoric correlation matrices with 5000 simulated datasets supported the retention of a single factor. A unidimensional model was subsequently extracted using the minimum residual (MinRes) method. Because only one factor emerged, factor rotation was not performed. All six items showed substantial factor loadings ranging from 0.648 to 0.900 (communalities from 0.420 to 0.811), providing further support for the unidimensional structure of the BSMAS-J (Table 2).

### 3.3. Confirmatory Factor Analysis (CFA) and Measurement Invariance

To cross-validate the factor structure on an independent dataset, a CFA was performed on the validation subsample (*n* = 214). The unidimensional model with six indicators and no correlated residuals was tested (*df* = 9). Robust maximum likelihood (MLR) estimation indicated good model fit overall: scaled *χ*^2^(9) = 11.22, *p* = 0.261, robust CFI = 0.995, robust TLI = 0.991, robust RMSEA = 0.043 (90% CI [0.000, 0.110]), and SRMR = 0.027. Standardized factor loadings were all statistically significant and substantial, ranging from 0.689 to 0.803 (all *p* < 0.001; Table 2). A sensitivity analysis using WLSMV estimation for ordinal data also indicated generally acceptable fit (robust CFI = 0.980, robust TLI = 0.966, SRMR = 0.028), although the robust RMSEA was 0.093 (90% CI [0.056, 0.133]), which should be interpreted in light of the small model degrees of freedom.

Measurement invariance across sex was evaluated in the full baseline sample (*N* = 314; 161 female, 153 male). As shown in Table 3, sequential equality constraints on thresholds, loadings, and intercepts resulted in minimal changes in comparative fit relative to the configural model (∆CFI ≥ −0.002, ∆SRMR ≤ 0.002), meeting standard invariance criteria, although the absolute RMSEA of the configural model was elevated (0.146). Longitudinal invariance was evaluated across the 4-week interval among paired participants (*n* = 257). After allowing correlated residuals between identical items across time, strict scalar invariance was supported, with negligible changes across configural, metric, and scalar models (∆CFI ≥ −0.002, ∆RMSEA ≤ 0.001, ∆SRMR < 0.001).

### 3.4. Reliability Analysis

The internal consistency of the BSMAS-J was high across both waves: at baseline (*N* = 314), Cronbach’s *α* was 0.90 and McDonald’s *ω* was 0.91 (corrected item–total correlations ranged from 0.63 to 0.76); at the 4-week follow-up (*n* = 257), Cronbach’s *α* was 0.89 and McDonald’s *ω* was 0.91 (corrected item–total correlations ranged from 0.65 to 0.77). Over the 4-week interval, test–retest reliability evaluated via a two-way mixed-effects, single-measurement, absolute-agreement intraclass correlation coefficient [ICC(A,1)] was 0.65 (95% CI [0.57, 0.71], *p* < 0.001). According to the guidelines of Koo and Li (2016), this value indicates moderate test–retest reliability.

### 3.5. Correlation and Regression Analysis

Zero-order Pearson correlation coefficients between the BSMAS-J and the validation instruments across the full baseline sample (*N* = 314) are presented in Table 4. Consistent with convergent validity, BSMAS-J scores showed a statistically significant positive correlation with the IAT (*r* = 0.60, *p* < 0.001). BSMAS-J scores also positively correlated with all behavioral activation and inhibition dimensions: BIS (*r* = 0.35, *p* < 0.001), BAS-D (*r* = 0.31, *p* < 0.001), BAS-FS (*r* = 0.29, *p* < 0.001), and BAS-RR (*r* = 0.22, *p* < 0.001). Regarding the TIPI-J Big Five personality dimensions, BSMAS-J scores were positively correlated with Neuroticism (*r* = 0.24, *p* < 0.001) and negatively correlated with Openness (*r* = −0.16, *p* = 0.004). The association with Extraversion was marginally negative (*r* = −0.11, *p* = 0.055), while the correlations with Agreeableness and Conscientiousness were not statistically significant.

To examine the multivariate associations of demographic, personality, and neurobehavioral traits on BSMAS-J scores, a hierarchical multiple linear regression analysis was performed (Table 5). Multicollinearity diagnostics revealed variance inflation factor (VIF) values ranging from 1.05 to 2.54, confirming the absence of problematic multicollinearity. The overall regression model accounted for 21.1% of the total variance in BSMAS-J scores (*R*^2^ = 0.211, *F*(11, 302) = 7.33, *p* < 0.001). In the simultaneous model using ordinary least squares (OLS), sex was a significant predictor (*b* = −1.22, *SE* = 0.60, *p* = 0.043), with male participants reporting lower BSMAS-J scores than female participants. In addition, both Neuroticism (*b* = 0.33, *SE* = 0.14, *p* = 0.019) and BIS (*b* = 0.29, *SE* = 0.10, *p* = 0.003) emerged as statistically significant positive predictors. When using heteroscedasticity-consistent standard errors (HC3), the association of BIS (*p* = 0.033) and Neuroticism (*p* = 0.041) remained robustly significant, whereas the association with sex was marginally significant (*p* = 0.051).

## 4. Discussion

The present study aimed to develop the Japanese version of the Bergen Social Media Addiction Scale (BSMAS-J) and evaluate its reliability, factor structure, measurement invariance, and convergent validity among Japanese college students. Overall, the findings supported the three pre-specified hypotheses and provided initial evidence regarding the psychometric properties of the BSMAS-J and its correlates among Japanese college students.

Regarding Hypothesis 1, both exploratory factor analysis (EFA) and confirmatory factor analysis (CFA) demonstrated that the BSMAS-J possesses a robust unidimensional factor structure, directly aligning with the original scale development (Andreassen et al., 2016). Standardized factor loadings were uniformly high, confirming that the six core addiction criteria effectively reflect a cohesive underlying construct.

Regarding Hypothesis 2, the scale exhibited high internal consistency across both waves (Cronbach’s *α* ≥ 0.89, McDonald’s *ω* ≥ 0.91). Evaluation of temporal stability across the 4-week interval yielded an ICC of 0.65, indicating moderate or adequate test–retest reliability according to standard psychometric benchmarks (Koo & Li, 2016). Furthermore, multigroup and longitudinal analyses supported measurement invariance. Longitudinal scalar invariance was established across the 4-week period, confirming that the measurement properties of the BSMAS-J were relatively stable over time. Multigroup analyses across sex demonstrated minimal changes in comparative fit indices (∆CFI ≤ 0.002). However, the elevated absolute RMSEA in the configural model suggests that measurement invariance across sex should be interpreted cautiously and examined further in larger samples.

Regarding Hypothesis 3, zero-order correlation analyses provided support for convergent construct validity. BSMAS-J scores showed a substantial positive correlation with generalized internet addiction (IAT), indicating that while the BSMAS-J captures platform-specific social networking vulnerabilities, it shares substantial common variance with broader online addictive behaviors (Andreassen et al., 2012, 2016; Leung et al., 2020). In addition, BSMAS-J scores showed significant correlations with neurobehavioral systems (BIS/BAS) and Big Five personality traits (TIPI-J), with patterns that were broadly consistent with those reported in previous studies (Andreassen et al., 2012, 2016; Wartberg et al., 2025).

When predictors were examined simultaneously in multiple regression analysis across the full baseline sample (*N* = 314), several additional patterns emerged. First, female sex was associated with higher BSMAS-J scores, consistent with the previous literature reporting higher levels of problematic social media addiction among females (e.g., Andreassen et al., 2012; Bányai et al., 2017; Su et al., 2020). One possible explanation is that differences in patterns and motivations for social media use may contribute to this association. Previous research suggests that females may be more likely to utilize social media for interpersonal interaction, relational maintenance, seeking social feedback, and managing online self-presentation (Twenge & Martin, 2020; Yau & Reich, 2019), as well as coping with emotional distress (Chae et al., 2018), thereby elevating the risk of excessive engagement.

Second, regarding neurobehavioral systems and personality traits, comparing the present predictive patterns with foundational validation research provides valuable theoretical insights (Andreassen et al., 2012). In the original development study of the Bergen Facebook Addiction Scale, approach-oriented neurobehavioral tendencies, specifically Fun Seeking (BAS-FS), emerged as a significant predictor, reflecting novelty seeking and reward-driven platform engagement (Andreassen et al., 2012). In contrast, within the present study, the Behavioral Inhibition System (BIS)—an avoidance-oriented trait—served as the sole statistically significant neurobehavioral predictor among the BIS/BAS dimensions. Although recent empirical evidence has emphasized that deficits in cognitive executive inhibitory control play a critical role in problematic social media use among emerging adults (Shannon et al., 2025), the BIS assessed herein captures dispositional sensitivity to punishment, threat, and negative social feedback rather than executive motor control (Carver & White, 1994; Kamide & Daibo, 2005).

Crucially, multiple regression analysis further revealed that Neuroticism emerged as a statistically significant positive predictor alongside BIS. The observed co-occurrence of high Neuroticism and elevated BIS sensitivity offers a coherent psychological profile: individuals with this dispositional configuration may experience greater anxiety in face-to-face interpersonal environments, prompting them to turn to social media as an avoidance-oriented coping strategy to manage dysphoric moods or escape offline social discomfort. While this avoidance- and affect-regulation-driven pattern presents an intriguing contrast to the approach-driven mechanisms observed in Western contexts (Andreassen et al., 2012), the single-country, cross-sectional design of the present study precludes inferring definitive cultural drivers or causal pathways. Rather, these findings should be considered exploratory empirical observations that provide concrete working hypotheses for future cross-cultural and longitudinal investigations.

Several limitations of the present study should be noted, pointing to important avenues for future research. First, the sample was restricted to undergraduate university students recruited via an online research panel (Freeasy Inc.), and individuals who used social media less than once a week were excluded from participation. Consequently, findings may be subject to self-selection bias and cannot be directly generalized to younger adolescents, non-student working adults, clinical populations, or infrequent users.

Second, the study relied entirely on self-report questionnaires, which introduces the possibility of common method bias, recall inaccuracies, and social desirability. Future investigations would benefit from incorporating objective behavioral tracking (e.g., smartphone screen-time logs) or structured clinical diagnostic interviews to establish external criterion validity.

Third, the observed predictive associations between BSMAS-J scores, personality traits, and neurobehavioral systems were based on cross-sectional data. The direction of these associations therefore cannot be determined. Longitudinal studies or experimental designs may help clarify the temporal and potentially causal relationships between these psychological characteristics and problematic social media use.

Finally, from a psychometric perspective, although split-sample cross-validation supported the unidimensional structure, the absolute RMSEA in multigroup configural models across sex was elevated. Given the sensitivity of RMSEA in models with small degrees of freedom (*df* = 9; Kenny et al., 2015), this finding should be interpreted alongside the other fit indices rather than in isolation. Nevertheless, further large-scale replications across diverse Japanese subpopulations and cross-national cohorts are warranted to further evaluate the measurement invariance and generalizability of the BSMAS-J.

## 5. Conclusions

The present study developed and validated the Japanese version of the Bergen Social Media Addiction Scale (BSMAS-J) among Japanese college students. Employing an independent split-sample cross-validation design, the BSMAS-J demonstrated a unidimensional structure, high internal consistency across waves, and adequate 4-week test–retest reliability. Evidence also supported longitudinal scalar invariance and provided preliminary support for measurement invariance across sex. Convergent validity was established via substantial positive associations with generalized internet addiction. In multiple regression analysis, female sex, higher neuroticism, and elevated behavioral inhibition were identified as statistically significant cross-sectional predictors of BSMAS-J scores. Overall, the BSMAS-J appears to be a concise and reliable instrument for assessing problematic social media use in Japan and provides a useful basis for future cross-cultural and longitudinal research.

## Figures and Tables

**Table 1:** Participant flow and demographic characteristics.

Domain	Characteristic	Baseline (*N* = 314)	Follow-Up (*n* = 257)
Sample flow	Initially recruited	363	—
	Excluded: not a college student	49	—
	Baseline analytic sample	314	—
	Completed 4-week follow-up	257	257
	Did not complete follow-up	57	—
Age, years	M (SD) [range]	20.44 (1.49) [18–24]	20.48 (1.52) [18–24]
Sex	Female	161 (51.3)	133 (51.8)
	Male	153 (48.7)	124 (48.2)
Marital status	Unmarried	314 (100.0)	257 (100.0)
Children	None	314 (100.0)	257 (100.0)
Residence	Owner-occupied detached house	152 (48.4)	122 (47.5)
	Owner-occupied condominium	42 (13.4)	35 (13.6)
	Rented apartment	51 (16.2)	42 (16.3)
	Rented condominium	43 (13.7)	36 (14.0)
	Rented detached house	6 (1.9)	6 (2.3)
	Dormitory or boarding house	7 (2.2)	4 (1.6)
	Company housing	2 (0.6)	2 (0.8)
	Other	11 (3.5)	10 (3.9)
Annual household income	<¥1.0 million	73 (23.2)	61 (23.7)
	¥1.0–<2.0 million	18 (5.7)	15 (5.8)
	¥2.0–<3.0 million	14 (4.5)	11 (4.3)
	¥3.0–<4.0 million	22 (7.0)	18 (7.0)
	¥4.0–<5.0 million	25 (8.0)	18 (7.0)
	¥5.0–<6.0 million	35 (11.1)	31 (12.1)
	¥6.0–<7.0 million	24 (7.6)	20 (7.8)
	¥7.0–<8.0 million	23 (7.3)	16 (6.2)
	¥8.0–<9.0 million	19 (6.1)	13 (5.1)
	¥9.0–<10.0 million	19 (6.1)	19 (7.4)
	¥10.0–<12.0 million	14 (4.5)	12 (4.7)
	¥12.0–<15.0 million	10 (3.2)	6 (2.3)
	¥15.0–<18.0 million	6 (1.9)	6 (2.3)
	¥18.0–<20.0 million	4 (1.3)	4 (1.6)
	≥¥20.0 million	8 (2.5)	7 (2.7)

Note. Values are *n* (%) unless otherwise indicated. Percentages are calculated within each analytic sample. The baseline sample contains all 314 eligible college students; no participant was excluded on the basis of the BSMAS-J total score.

**Table 2:** Item-level statistics and factor loadings for the BSMAS-J at baseline.

Item	Component	*M*	*SD*	Skewness	Kurtosis	EFA λ	CFA λ	Corrected Item–Total r
BSMAS-J-1	Salience	3.19	1.09	−0.21	−0.52	0.648	0.689	0.63
BSMAS-J-2	Tolerance	2.85	1.18	0.06	−0.87	0.879	0.772	0.75
BSMAS-J-3	Mood modification	2.80	1.24	0.12	−0.95	0.825	0.747	0.72
BSMAS-J-4	Relapse	2.69	1.21	0.15	−0.97	0.900	0.781	0.76
BSMAS-J-5	Withdrawal	2.67	1.18	0.16	−0.91	0.865	0.803	0.76
BSMAS-J-6	Conflict	2.59	1.16	0.24	−0.76	0.836	0.793	0.74

Note. BSMAS-J = Japanese Bergen Social Media Addiction Scale; EFA = exploratory factor analysis; CFA = confirmatory factor analysis. EFA used polychoric correlations and minimum residual extraction in an EFA subsample (*n* = 100). CFA loadings are standardized MLR estimates from the independent CFA subsample (*n* = 214).

**Table 3:** Confirmatory factor analysis and measurement invariance fit indices.

Analysis	Model	*n*	Scaled *χ*^2^	*df*	*p*	CFI	TLI	RMSEA [90% CI]	SRMR	ΔCFI	ΔRMSEA	ΔSRMR
Independent CFA	One factor	214	11.22	9	0.261	0.995	0.991	0.043 [0.000, 0.110]	0.027			
Sex invariance	Configural	314	78.04	18	<0.001	0.983	0.972	0.146 [0.114, 0.180]	0.040			
	Thresholds	314	81.42	30	<0.001	0.985	0.985	0.105 [0.078, 0.132]	0.040	0.002	−0.041	−0.000
	Metric	314	93.19	35	<0.001	0.983	0.986	0.103 [0.078, 0.129]	0.042	−0.002	−0.002	0.002
	Scalar	314	101.03	40	<0.001	0.983	0.987	0.099 [0.075, 0.123]	0.042	−0.001	−0.004	−0.000
Longitudinal invariance	Configural	257	119.99	47	<0.001	0.984	0.977	0.078 [0.061, 0.095]	0.035			
	Thresholds	257	131.59	59	<0.001	0.984	0.982	0.069 [0.053, 0.085]	0.035	0.000	−0.009	−0.000
	Metric	257	139.35	64	<0.001	0.983	0.983	0.068 [0.052, 0.083]	0.036	−0.001	−0.002	0.001
	Scalar	257	152.73	69	<0.001	0.981	0.982	0.069 [0.054, 0.084]	0.036	−0.002	0.001	0.000

Note. The independent CFA treated the five response categories as approximately continuous and used MLR; robust CFI, TLI, and RMSEA are reported for this model. Measurement-invariance models treated the responses as ordered and used WLSMV with theta parameterization; their incremental fit indices are scaled. Changes are relative to the preceding model within each invariance sequence. Although changes in CFI, RMSEA, and SRMR were small, the elevated absolute RMSEA for the sex-configural model warrants cautious interpretation.

**Table 4:** Descriptive statistics, reliability, and correlations among study variables.

Variable	*M*	*SD*	Reliability	1	2	3	4	5	6	7	8	9	10
1. BSMAS-J	16.78	5.75	α = 0.90										
2. IAT	50.83	16.72	α = 0.95	0.60 ***									
3. BIS	17.02	4.28	α = 0.78	0.35 ***	0.61 ***								
4. BAS-RR	18.55	4.65	α = 0.86	0.22 ***	0.32 ***	0.58 ***							
5. BAS-D	7.17	2.26	α = 0.77	0.31 ***	0.47 ***	0.55 ***	0.53 ***						
6. BAS-FS	7.57	2.19	α = 0.75	0.29 ***	0.40 ***	0.54 ***	0.67 ***	0.67 ***					
7. Extraversion	6.93	2.80	rSB = 0.62	−0.11	0.03	−0.06	−0.00	0.06	0.07				
8. Neuroticism	8.46	2.42	rSB = 0.50	0.24 ***	0.20 ***	0.26 ***	0.15 **	0.16 **	0.05	−0.34 ***			
9. Openness	7.66	2.46	rSB = 0.52	−0.16 **	−0.09	−0.15 **	−0.01	0.05	0.10	0.31 ***	−0.24 ***		
10. Agreeableness	9.02	2.24	rSB = 0.33	0.01	−0.13 *	−0.10	0.09	−0.11 *	−0.00	−0.17 **	−0.01	−0.04	
11. Conscientiousness	7.82	2.43	rSB = 0.54	−0.05	−0.20 ***	−0.19 ***	−0.12 *	−0.07	−0.07	0.23 ***	−0.25 ***	0.19 ***	0.15 **

Note. *N* = 314. Values below the diagonal are Pearson correlations. Reliability is Cronbach’s alpha for multi-item scales and the Spearman–Brown coefficient (rSB) for the two-item TIPI-J dimensions. For the BSMAS-J, McDonald’s *ω* was 0.91. TIPI-J scores were recomputed after reverse-keying the designated items. * *p* < 0.05. ** *p* < 0.01. *** *p* < 0.001.

**Table 5:** Hierarchical regression predicting BSMAS-J scores.

Model	Predictor	*b*	*SE*	95% CI	*β*	*t*	*p*
Model 1	Intercept	23.97	4.41	[15.28, 32.65]		5.43	<0.001
	Age	−0.31	0.22	[−0.73, 0.12]	−0.08	−1.43	0.154
	Sex (male = 1)	−1.85	0.64	[−3.11, −0.59]	−0.16	−2.88	0.004
Model 2	Intercept	12.38	4.90	[2.73, 22.02]		2.53	0.012
	Age	−0.25	0.20	[−0.65, 0.14]	−0.07	−1.25	0.211
	Sex (male = 1)	−1.22	0.60	[−2.41, −0.04]	−0.11	−2.03	0.043
	Extraversion	−0.07	0.12	[−0.31, 0.17]	−0.03	−0.58	0.560
	Neuroticism	0.33	0.14	[0.05, 0.60]	0.14	2.36	0.019
	Openness	−0.24	0.13	[−0.50, 0.02]	−0.10	−1.82	0.069
	Agreeableness	0.06	0.14	[−0.21, 0.34]	0.02	0.45	0.654
	Conscientiousness	0.15	0.13	[−0.10, 0.41]	0.07	1.18	0.240
	BIS	0.29	0.10	[0.10, 0.48]	0.22	3.02	0.003
	BAS-RR	−0.13	0.09	[−0.31, 0.06]	−0.10	−1.35	0.179
	BAS-D	0.33	0.19	[−0.04, 0.70]	0.13	1.76	0.080
	BAS-FS	0.40	0.21	[−0.02, 0.82]	0.15	1.86	0.064

Note. *N* = 314. Model 1: *R*^2^ = 0.033, adjusted *R*^2^ = 0.027, *F*(2, 311) = 5.27, *p* = 0.006. Model 2: *R*^2^ = 0.211, adjusted *R*^2^ = 0.182, *F*(11, 302) = 7.33, *p* < 0.001. Δ*R*^2^ = 0.178, *F*change(9, 302) = 7.56, *p* < 0.001. Sex was coded 0 = female and 1 = male. Coefficients are cross-sectional associations and do not establish causal drivers.

## Data Availability

The datasets used during the current study are available from the corresponding author.

## Supplementary Materials

The following supporting information can be downloaded at: https://www.mdpi.com/article/10.3390/ejihpe16100148/s1, The Japanese version of the Bergen Social Media Addiction Scale (BSMAS-J).
