# Supplementary material for: Development and Validation of the Japanese Version of the Bergen Social Media Addiction Scale (BSMAS-J)
Source: Eur J Investig Health Psychol Educ. 2026 Sep 24;16(10):148. doi: 10.3390/ejihpe16100148 (PMC13703265; doi:10.3390/ejihpe16100148)
Supplement: Supplementary file 1 [file ejihpe-16-00148-s001.zip › ejihpe-4571356-supplementary.pdf]

日本語版ソーシャルメディア依存傾向尺度（BSMAS-J）、6 項目、5 件法

説明文：以下の質問は、あなたとソーシャルメディア（Facebook, Twitter, Instagram, LINE, TikTok, YouTube など）の関係や利用に関するものです。各質問について、最も適切な選択肢を選んでください。

過去 1 年間において、あなたはどのくらいの頻度で…　「ほとんどない・少ない・時々・しばしば・非常に多い」

1) …ソーシャルメディアを頻繁に気にかけたり、ソーシャルメディアを使おうと考えたりしましたか？
2) …ソーシャルメディアをもっと多く使いたいという衝動を感じましたか？
3) …個人が抱える問題を忘れるために、ソーシャルメディアを使用しましたか？
4) …ソーシャルメディアの使用を減らそうと試みたが、うまくいきませんでしたか？
5) …ソーシャルメディアの使用が禁止された場合に、落ち着かなくなったり、不安になったりしましたか？
6) …ソーシャルメディアをあまりにも多く使用したために、仕事や勉強に悪影響を与えましたか？

英語対応

1. ソーシャルメディアを頻繁に気にかけたり、ソーシャルメディアを使おうと考えたりしましたか

1. Spent a lot of time thinking about social media or planned use of social media

2. ソーシャルメディアをもっと多く使いたいという衝動を感じましたか

2. Felt an urge to use social media more and more

3. 個人が抱える問題を忘れるために、ソーシャルメディアを使用しましたか

3. Used social media in order to forget about personal problems

4. ソーシャルメディアの使用を減らそうと試みたが、うまくいきませんでしたか

4. Tried to cut down on the use of social media without success

5. ソーシャルメディアの使用が禁止された場合に、落ち着かなくなったり、不安になったりしましたか

5. Become restless or troubled if you have been prohibited from using social media

6. ソーシャルメディアをあまりにも多く使用したために、仕事や勉強に悪影響を与えましたか

6. Used social media so much that it has had a negative impact on your job/studies
